# Cost-savings and potential cost-savings through the distribution of generic antiretroviral drugs within the statutory health insurance market of Germany between January 2017 and June 2019

**DOI:** 10.1186/s12913-021-07390-4

**Published:** 2022-01-13

**Authors:** Matthäus Lottes, Viviane Bremer, Christof Prugger, Christian Kollan, Daniel Schmidt

**Affiliations:** 1 Department of Methodology and Research Infrastructure, Information and Research Data Management, Robert Koch Institute, Berlin, Germany; 2 Department of Infectious Disease Epidemiology, HIV/AIDS, STI and Blood-borne Infections, Robert Koch Institute, Berlin, Germany; 3 Institute of Public Health, Charité - Universitätsmedizin, Berlin, Germany

**Keywords:** Generic antiretroviral therapy, German statutory health insurance, Tenofovir disoproxil/emtricitabine, Tenofovir alafenamide/emtricitabine, Cost-saving, Potential cost-saving

## Abstract

**Abstract:**

**Background:**

Recent patent losses for antiretroviral drugs (ARV) have led to the debate of cost-saving through the replacement of patented drugs with generic drugs. The split of recommended single-tablet regimens (STR) into their single substance partners is one of the considerations mentioned in said debate. Particularly, generic tenofovir disoproxil/emtricitabine (TDF/FTC) is expected to hold untapped cost-saving potential, which may curb increasing overall expenditures for combined antiretroviral therapy (cART) within the statutory health insurance (SHI) of Germany.

**Methods:**

Data of ARV reimbursed by the SHI were used to describe the trends of defined daily doses (DDD) as well as the revenue within the German ARV market. They were also used to determine the cost-savings of moving to generic drugs. The time period observed was between January 2017 and June 2019. The potential cost-savings were determined with following assumption in mind: the maximum possible use of generic ARV, including 1) the split of STR and replacing all substance partners with generic ones, and 2) replacing patented tenofovir alafenamide/emtricitabine (TAF/FTC) with generic TDF/FTC.

**Results:**

Throughout the observation period, the DDD of generic ARV increased nearly five-fold while their revenue increased more than four-fold. Total cost-saving showed a sharp increase over the same period, with generic TDF/FTC accounting for a share of around 70%. The largest potential cost-saving could have been achieved through replacing patented TAF/FTC with generic TDF/FTC, peaking at nearly 10% of total revenue, but showing decreasing trends in general.

**Conclusion:**

The progressive distribution of generic ARV ensured increasing cost-savings_,_ but consequently curbed the potential cost-savings. Unique price reductions of generic TDF/FTC have played a pivotal role for these effects. In any case, substituting with generic ARV should not fail to adhere to the treatment guidelines and continue to consider the medical requirements for the treatment.

**Supplementary Information:**

The online version contains supplementary material available at 10.1186/s12913-021-07390-4.

## Background

Combined antiretroviral therapy (cART) has substantially reduced the morbidity and mortality of the growing number of people infected with human immunodeficiency virus (HIV) [1–3]. HIV treatment in Germany has been very successful, with 93% of treated persons virally suppressed [4]. Therefore, cART has been the standard of care since its introduction in the mid-1990s and became the highest direct cost of HIV treatment in Germany, accounting for more than 85% of costs [5–7]. While the total expenditures for all drugs within the statutory health insurance (SHI) of Germany amounted to approximately 39 billion € in 2018 [8], the expenditures on antiretroviral drugs (ARV) for patients with HIV, representing less than 1% of the total health insured community of Germany, amounted to approximately 1.3 billion € (≈3% of total drug expenditures) [9, 10].

Important regulations have been introduced by the German legislation with the aim of curbing prices of patented drugs as well as the increasing expenditures on pharmaceuticals incurred by sickness funds. These include, firstly, the German Act of reinforcing SHI competition that was passed in 2007 in order to facilitate the use of generic drugs after the patent expiration of branded drugs which is usually 20 years after the filings of patent applications [11–14]. Secondly, the Act on the Reform of the Market for Medical Products, passed in 2011, which obligated manufacturers to demonstrate the additional therapeutic benefit of newly developed pharmaceuticals. This additional benefit is the basis for the price negotiations between the Federal Association of Statutory Health Insurance Funds and pharmaceutical manufacturers [15, 16]. Furthermore, as per the Social Code Book, German sickness funds are permitted to negotiate discount agreements with drug manufacturers [17].

Regardless of economic interests, the distribution of ARV must adhere to current treatment guidelines that recommend a combination of three drug classes for the initiation of the HIV therapy, preferably as a single-tablet regimen (STR) [18]. Generic ARV started to gain popularity in Germany between 2011 and 2013, starting with zidovudine, and followed by lamivudine, nevirapine and efavirenz [19]. The initial proportion of generic ARV in 2014 was rather small with 3.1% of total ARV distribution. When assuming the maximum possible use of generic ARV, the total potential cost-saving would have been 8% [20]. However, according to current treatment guidelines, most of these substances are no longer recommended for the initiation of the antiretroviral therapy, causing their market share to decline [5, 18]. In contrast, the launch of generic tenofovir disoproxil/emtricitabine (TDF/FTC) in August 2017 brought about a transformative change to the market of generic prescriptions. This was driven by its approval for pre-exposure prophylaxis, leading to unique price reduction of more than 90% [21], whereas price reductions for generic drugs within the German ARV market usually range between 10 and 40% [22]. Subsequently, a high budget impact was expected, as more than 70% of the cART regimens in Germany were based on TDF/FTC in 2017 [23].

Since almost one-third of the available ARV lost their patent protections, the debate about cost-saving through generic replacements is still ongoing [5, 24–27]. This entails the split of newer and recommended STR into their single substance partners, irrespective of the medical indication for a change of treatment and accepting the possible consequences of higher pill burdens for patients [28–30].

In this study, our aim was to firstly describe the current ARV market within the German SHI. Secondly, we aimed to determine the cost-savings achieved through the distribution of generic drugs, focusing particularly on TDF/FTC. Our third aim was to determine the potential cost-savings that could be achieved additionally when assuming the maximum possible use of generic ARV. This includes the splitting of STR, replacing all substance partners with generic ones and replacing the patented drug tenofovir alafenamide/emtricitabine (TAF/FTC) with generic TDF/FTC.

## Methods

### Data source

Insight Health™ provided the data of distributed ARV for the years 2013 to the end of the second quarter of 2019. These data were collected from the billing centers and included all prescriptions reimbursed to pharmacies by the German SHI. The data account for more than 99% of distributed ARV covered by the SHI.

### Data structure and editing

The dataset included all ARV by pharmaceutical registration numbers distributed on a monthly basis, regardless of whether the use was for prevention or treatment of HIV. Additional relevant items were product name, substance name, drug class, Anatomical Therapeutic Chemical (ATC) classification code which is unique for each ARV and thus will be used synonymously throughout the paper [31], patent status, pharmacy retail price and defined daily dose (DDD). DDD was determined as the daily per capita consumption for each antiretroviral substance grouped by ATC, based on manufacturer specifications and guidelines [18, 32]. The ATC codes considered are listed by substance class, antiretroviral substances and quarter/year of launch as a generic drug (Table 1). For the sake of clarity, two items were excluded from the analysis: 1) ARV that were not available in tablet form and 2) the DDD of single TDF mainly used for the treatment of hepatitis B [33–35]. The DDD of the booster substances ritonavir and cobicistat were quantified by counting the DDD of the necessary combined protease inhibitor, ATC class J05AE (Table 1), and their recommended boosting doses [18, 36].

**Table 1:**
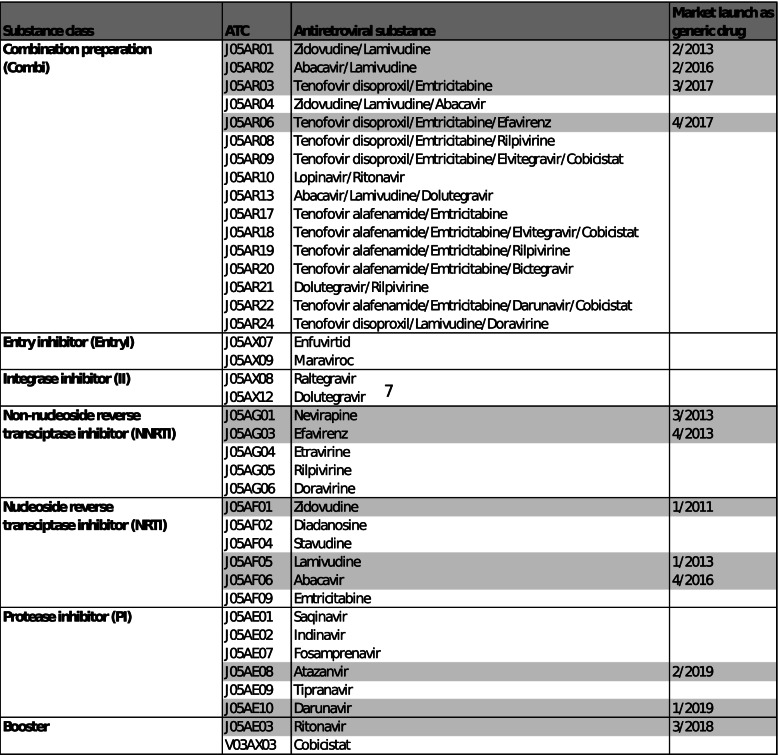
Anatomical Therapeutic Chemical (ATC) classifications by substance class, antiretroviral substances and quarter/year of launch as a generic drug in Germany (grey: available as generic drug)

### Determination of cost-savings and potential cost-savings

The observational period was limited from January 2017 to June 2019 in order to account for market launches of generic alternatives. For all ARV, the DDD and corresponding revenues were determined in accordance with their respective patent status: patented, patent expired or generic. Additionally, the DDD and revenues were determined for a few ARV with an expired patent that do not have an equivalent generic substitute.

To quantify cost-savings and potential cost-savings, the pharmacy retail price for each ARV was first calculated as a weighted average of patent status (Supplementary Table S1). Secondly, the exclusive distribution of patented or expired patent ARV has been assumed in order to determine cost-savings of moving to generic drugs. Thirdly, the maximum possible use of generic ARV has been assumed as explained before in order to determine the potential cost-savings. This includes the splitting of STR, replacing all substance partners with generic ones and replacing patented TAF/FTC with generic TDF/FTC.

## Results

**Fig. 1:**
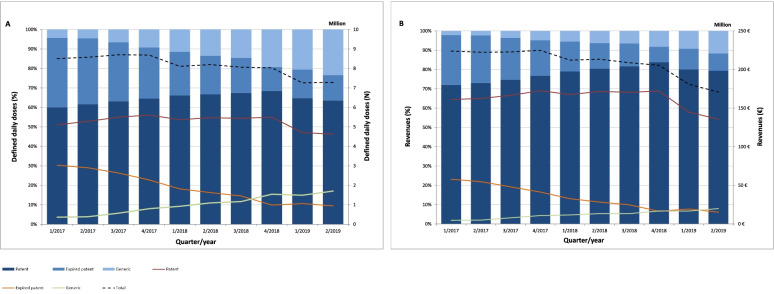
Defined daily doses (N, %) and revenues (million €, %) of antiretroviral drugs by patent status between 1/2017 and 2/2019

From 1/2017 to 2/2019, the DDD of generic ARV increased nearly five-fold and their revenue by more than four-fold (Fig. 1 A and B). The DDD of ARV with an expired patent, on the other hand, decreased continuously, while patented ARV showed a sharp decrease for the first time in 2019. In total, DDD and revenue decreased stepwise by one-sixth and by almost one-fourth, respectively.

**Fig. 2:**
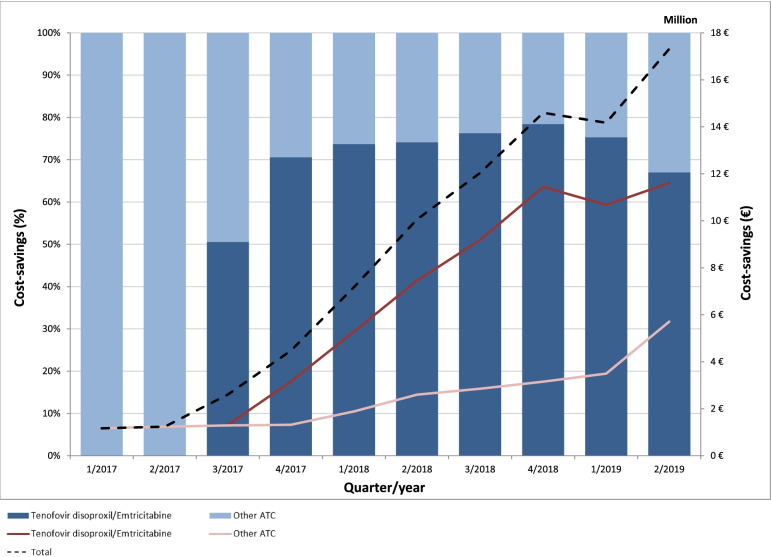
Cost-savings (million €, %) through the distribution of generic antiretroviral drugs between 1/2017 and 2/2019

The total cost-saving through the distribution of generic ARV showed a steep increase from 1.2 million € (< 1% of total revenues) in 1/2017 to 17.3 million € (10% of total revenues) in 2/2019 (Fig. 2). TDF/FTC leveled off at about 70% from 4/2017 until 4/2018, and the decline in its cost-savings thereafter may be attributed to the launch of generic darunavir (DRV) in 1/2019 (Table 1). A detailed overview of the cost-savings achieved by each generic ARV can be found in Supplementary Table S2.

**Fig. 3:**
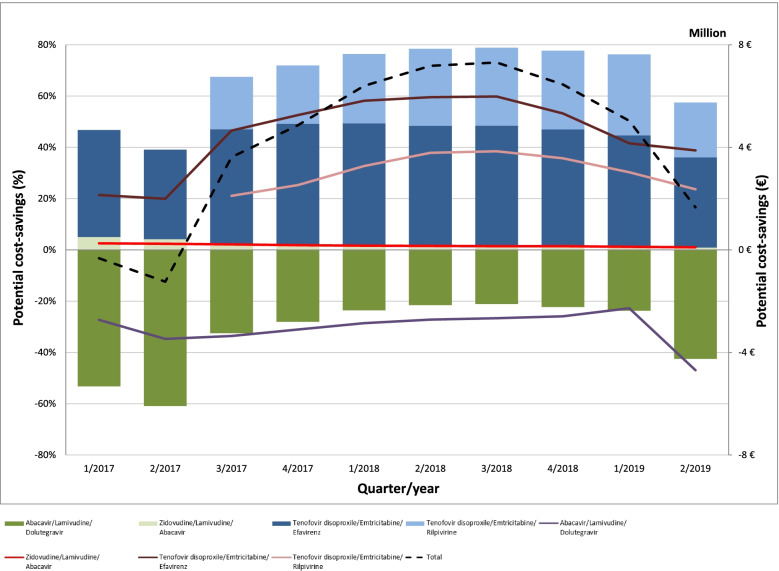
Potential cost-savings (million €, %) through the splitting of single-tablet regimens and replacing all substance partners with generic antiretroviral drugs between 1/2017 and 2/2019

**Fig. 4:**
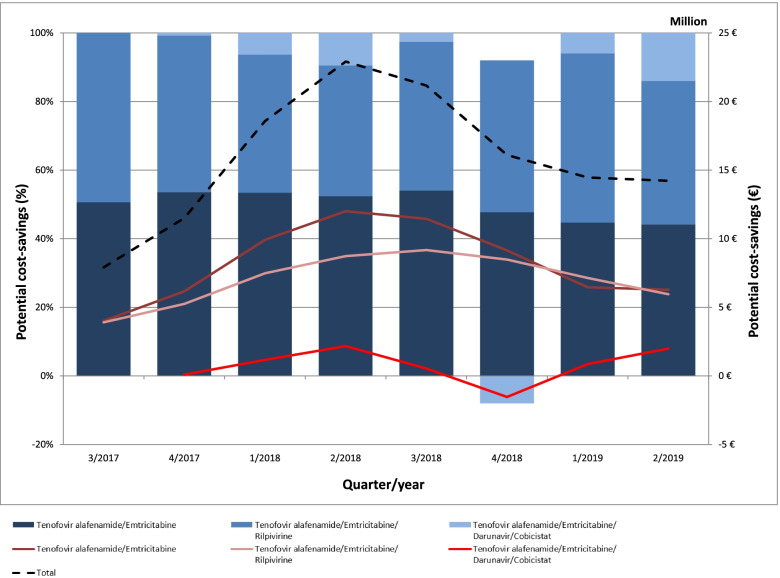
Potential cost-savings (million €, %) through replacing patented tenofovir alafenamide/emtricitabine with generic tenofovir disoproxil/emtricitabine between 3/2017 and 2/2019

Generic TDF/FTC also has a pivotal influence on the course of potential cost-savings, particular for the splitting and generic replacement of tenofovir disoproxil/emtricitabine/rilpivirine and tenofovir disoproxil/emtricitabine/efavirenz (Fig. 3) as well as for TAF/FTC and tenofovir alafenamide/emtricitabine/rilpivirine (Fig. 4). However, maximizing generic substitution would not have led consistently into potential cost-savings and overall downward trends have been observed since mid-2018. A detailed overview of the potential cost-savings of each ARV can be found in Supplementary Tables S3, S4, S5. Accordingly, the combination of potential cost-savings through a maximum possible use of generic ARV increased to 38.3 million € in 2/2018 (18% of total revenues) and then decreased to 19.2 million € in 2/2019 (11% of total revenues).

## Discussion

Using data of ARV reimbursed by the SHI, this study shows the increasing influence of generic ARV in Germany, accounting for almost one-fourth of the total ARV market in mid-2019. In particular, the unique price reductions for generic TDF/FTC substantially affected the course of cost-savings and potential cost-savings between January 2017 and June 2019. Assuming the maximum possible use of generic ARV, the splitting of STR and replacing TAF/FTC with generic TDF/FTC specifically, revealed substantial potential cost-savings.

### Market situation of antiretroviral drugs

The results showed a remarkable increase of distributed generic DDD (Fig. 1) as a result of the efforts of German sickness funds aiming to compensate the increasing cART expenditures by distributing generic ARV [17, 24]. However, our data suggested insufficient generic substitution for ARV with an expired patent, such as zidovudine, lamivudine or abacavir (Supplementary Table S1).

Discount agreements between sickness funds and drug manufacturers are an important factor to consider as they are binding for the distributing pharmacies [14, 37, 38]. However, as our calculations were solely based on the pharmacy retail price, we were not able to explicitly quantify the amounts of discount agreements. One may roughly approximate the discount agreements for ARV with an expired patent to be in the comparable range as the cheaper pharmacy retail price of their equivalent generic drugs.

Against the widespread use of generic ARV, the current guidelines recommend the treatment with patented STR [18], leading into their increased distribution (Supplementary Table S1) and, in turn, the observed decline of overall DDD (Fig. 1A).

### Cost-savings through the distribution of generic antiretrovirals

This study emphasizes the significant influence of generic TDF/FTC for cost-saving within the German ARV market, accounting for a share of approximate 70% (Fig. 2), triggered by its immense price drop in combination with the approval for the pre-exposure prophylaxis [21, 23]. The distribution of other generic ARV, on the other hand, accounted for a much smaller share of total cost-savings (Supplementary Table S1). This may certainly also have been due to their comparatively lower price reduction of between 10 and 40% [22, 23]. Overall, the growing importance of distributing generic ARV was confirmed, considering the strong increase of the total cost-saving from less than 1% (≈1.2 million €) of total ARV evenue in early 2017, to about 10% (≈17.3 million €) in mid-2019 (Supplementary Table S2).

### Potential cost-savings through the maximum possible use of generic antiretrovirals

Generic TDF/FTC also had a decisive role when assuming the maximum possible use of generic drugs in order to determine the potential cost-savings within the German ARV market. This mainly related to the split of STR (Fig. 3) and the replacement of patented TAF/FTC (Fig. 4). However, splitting STR into their single substance partners and replacing them with generic ARV did not guaranteed potential cost-savings, as observed for tenofovir disoproxil/lamivudine/doravirine and abacavir/lamivudine/dolutegravir (Fig. 3).

Moreover, this approach goes against the general trend of antiretroviral therapy [39–42] and implies a higher pill burden for patients who are used to taking STR, which might lower their treatment adherence [29, 30]. One should further note, that patented TAF/FTC is still recommended as preferred option for the initiation of antiretroviral therapy [18], which has shown superior renal and bone safety compared to TDF/FTC [43–45]. Therefore, a change of treatment routine should not solely focus on the potential cost-savings but also on medical concerns, such as acute toxicities or treatment failures [46, 47].

Finally, this study shows the declining potential cost-savings within the German ARV market, assuming the maximum possible use of generic drugs. Following this assumption, the total potential cost-savings would account for a rounded 11% (≈19.2 million €) of total ARV revenue in mid-2019 (Supplementary Table S3, S4, S5).

## Limitations

This paper has several limitations. Firstly, the dataset used did not allow access to the private health insurance sector which accounted for approximately 11,5% of the total health insured community of Germany [10]. This may have given a more complete picture.

Secondly, our assumption to determine the potential cost-savings was not applicable for ARV containing elvitegravir, bictegravir and lopinavir, since these substance partners are only available as a fixed combination preparation without generic substitute [18]. Since single TDF, assumed for the treatment of hepatitis B, was excluded, the split of tenofovir disoproxile/lamivudine/doravirine was also not considered (Table 1) [35].

Thirdly, we were informed that some pharmacies refused to transfer their data to the responsible billing centres in the health insurance regions of Berlin, North Rhine-Westphalia and Baden-Wuerttemberg at the end of 2018. This may have resulted in an additional decrease of total DDD, estimated at about 5–7%, in 2019.

## Conclusion

Generic drugs are progressively distributed in the German ARV market and ensure increasing cost-savings. Unique price reductions of generic TDF/FTC have played a pivotal role for both the cost-savings and potential cost-savings. However, as mentioned before, pursuing the maximum possible use of generic ARV should not fail to adhere to the treatment guidelines and consider the medical requirements for a change in treatment.

## Supplementary Information

**Additional file 1..**

## Data Availability

The dataset analyzed in this study is not publicly available, but can be made available upon reasonable request by Matthäus Lottes.

## Abbreviations

ARV: Antiretroviral drug
ATC: Anatomical Therapeutic Chemical classification
cART: Combined antiretroviral therapy
DDD: Defined daily dose
FTC: Emtricitabine
HIV: Human immunodeficiency virus
SHI: Statutory health insurance
STR: Single-tablet regimen
TAF: Tenofovir alafenamide
TDF: Tenofovir disoproxil
