# Supplementary material for: Cost-savings and potential cost-savings through the distribution of generic antiretroviral drugs within the statutory health insurance market of Germany between January 2017 and June 2019
Source: BMC Health Serv Res. 2022 Jan 13;22:63. doi: 10.1186/s12913-021-07390-4 (PMC8756633; doi:10.1186/s12913-021-07390-4)

Supplementary material

Table S1: Anatomical Therapeutic Chemical classifications with their defined daily dose (N), revenue (Euro), weighted average price (Euro) by patent status between 1/2017 and 2/2019


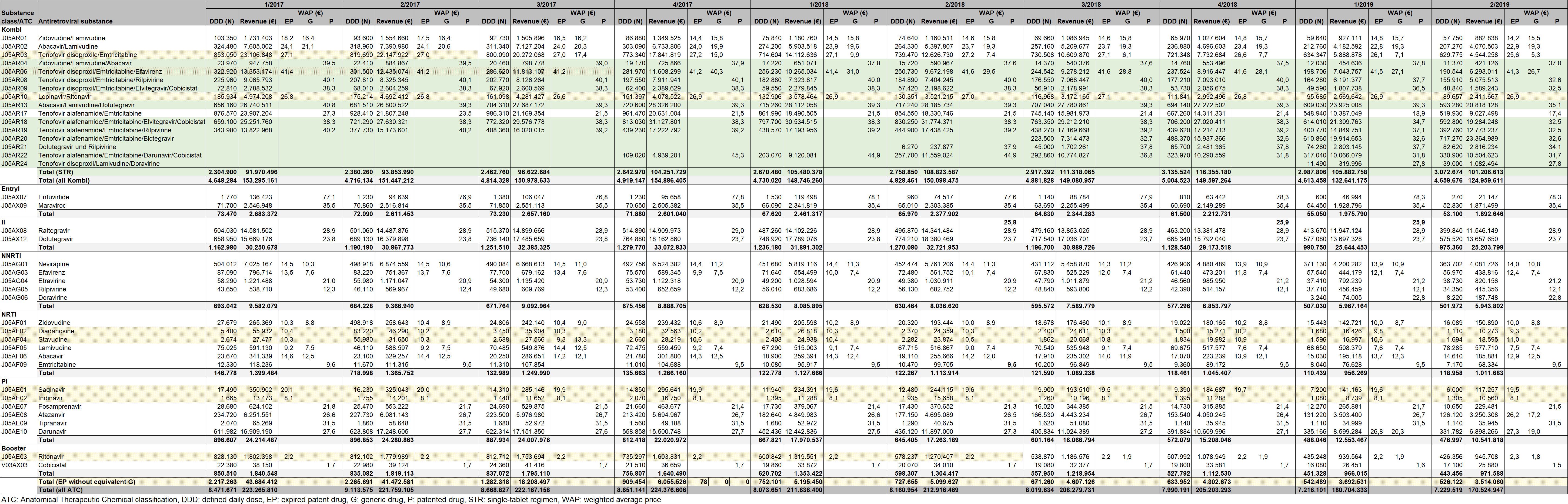


Table S2: Cost-savings (Euro, % of revenue) through the distribution of generic antiretroviral drugs between 1/2017 and 2/2019


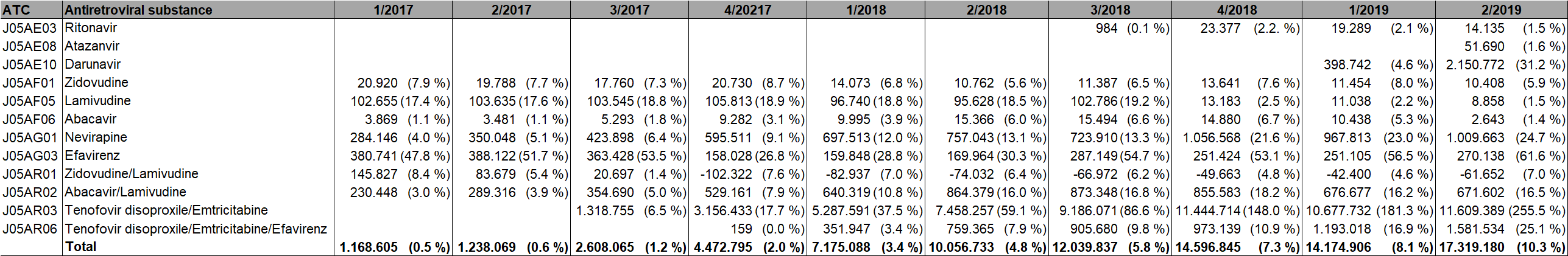


Table S3: Potential cost-savings (Euro, % of revenue) through the maximum possible use of generic antiretroviral drugs between 1/2017 and 2/2019


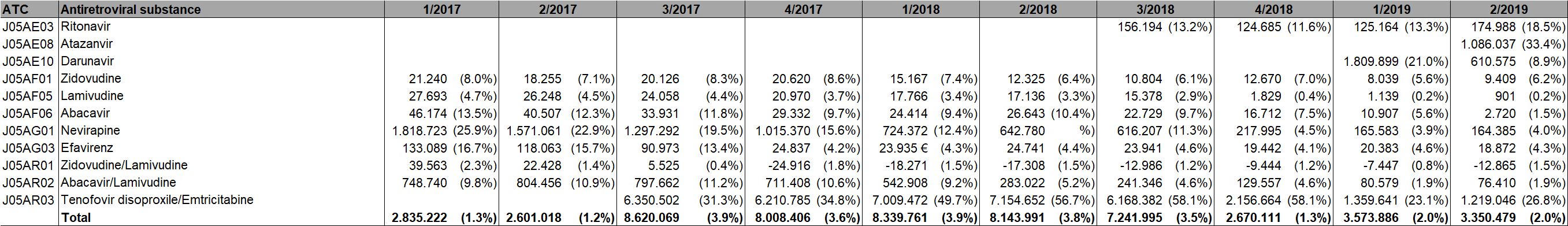


Table S4: Potential cost-savings (Euro, % of revenue) through the splitting of single-tablet regimens and replacing all substance partners with generic antiretroviral drugs between 1/2017 and 2/2019


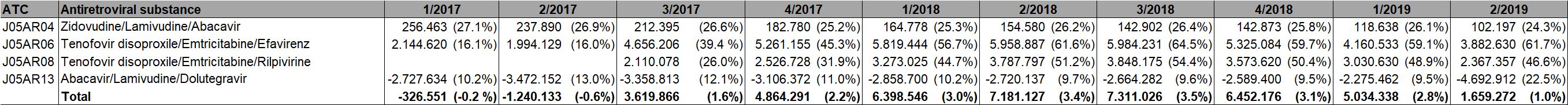


Table S5: Potential cost-savings (Euro, % of revenue) through replacing patented tenofovir alafenamide/emtricitabine with generic tenofovir disoproxil/emtricitabine between 1/2017 and 2/2019


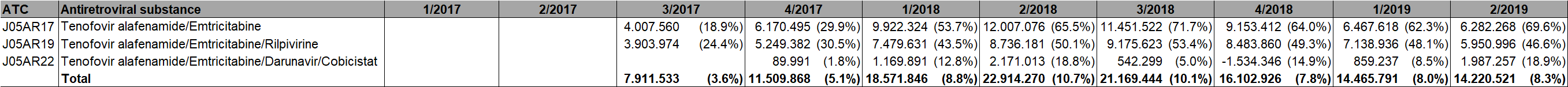

Supplement: Supplementary file 1 — Additional file 1.. [file 12913_2021_7390_MOESM1_ESM.docx]
